# Knowledge, Use, and Perception of Brazilian Women about Contraceptive Methods: An Observational Study

**DOI:** 10.1089/whr.2023.0185

**Published:** 2024-05-27

**Authors:** Juliana Dineia Perez Brandão, Rogerio Bonassi Machado, Ana Carolina Ferreira Cardoso

**Affiliations:** ^1^ Scientific Medical Division, Libbs Farmacêutica Ltda, São Paulo, Brazil.; ^2^ Department of Gynecology and Obstetrics, Jundiai School of Medicine, Jundiai, Brazil.

**Keywords:** contraceptive methods, survey, Brazilian women, family planning, socioeconomics of women health

## Abstract

**Background:**

In Brazil, where approximately 48.7 million women are of reproductive age, understanding contraceptive practices is essential for addressing public health challenges. This study evaluated into the knowledge, usage, and perceptions of contraceptive methods among Brazilian women, highlighting the influence of socioeconomic and demographic factors on their choices.

**Methods:**

We conducted a cross-sectional survey with a representative sample of 2000 Brazilian women aged 18–49 years. The questionnaire collected detailed information on their awareness, preferences, and utilization of various contraceptive methods, alongside demographic and socioeconomic data.

**Results:**

Oral contraceptives, condoms, injectables, and intrauterine devices (IUDs) were the most recognized methods. Younger women demonstrated greater awareness of modern methods. Socioeconomic disparities were evident, with lower-income women displaying limited knowledge about condoms and IUDs but a higher usage for injectable contraceptives. Oral contraceptives were the most used method, with higher use in the South, and lower in the Central-West and Northeast regions. Satisfaction with current contraceptive methods was high (87.5%), closely associated with personal responsibility in method choice. Although the majority self-financed their contraceptives (63.1%), a significant portion of lower-income women (27.7%) relied on public health care. Physicians’ recommendations predominantly influenced contraceptive choice (53.9%), with younger women also guided by other influences.

**Conclusions:**

Persistent disparities in contraceptive awareness and access highlight the need for educational initiatives and policy interventions. Health care providers play a vital role in facilitating informed contraceptive choices, enhancing the chances of satisfaction with the method.

## Introduction

Knowledge about contraceptive methods enables individuals to choose the most appropriate method for their health conditions and sexual behavior.^1^

Reversible methods include natural, barrier, chemical, and hormonal methods, whereas irreversible methods are surgical.^2,3^

In 2013, a study showed that 99% of women in the United States who have ever had sexual intercourse used at least one contraceptive method at some point in their lives, and the method used varied by factors such as race, education, and religious affiliation.^4^ In addition, patterns of contraceptive use vary across world regions, which may be related to demographics and access, such as differences in public policies that provide free or low-cost contraceptive methods, especially for low-income populations.^1^

In Latin America, although there has been a general decline in birth rates, unintended pregnancy remains prevalent.^5^ Previous data on Brazilian women of reproductive age found that 82.4% of them use contraception, mainly oral contraceptives (34.2%), followed by surgical methods (25.9%) and condoms (14.5%).^6^ Younger women made more use of oral contraceptives and condoms than older women, who were more likely to use intrauterine devices (IUDs). Contraceptive pills were used by at least 35% of women residing in the South and Southeast regions, as opposed to less than 20% of women living in the North, a less urbanized region. Surgical methods were more prevalent in rural areas and in women with less than 4 years of education.^6^

It is essential to consider individual preferences when implementing contraceptive intervention programs since dissatisfaction and side effects can affect adherence, increase discontinuation rates, and thus the risk of unintended pregnancy.^4,7,8^

Understanding potential variations in knowledge and use of contraceptive methods among distinct sociodemographic profiles could support the development of health policies and access programs that enhance informed decision making and adoption of the preferred methods to achieve reproductive autonomy.^1^ This study assessed the perceptions and awareness of contraceptive methods among Brazilian women of different sociodemographic backgrounds and identified the used methods and the factors influencing their choice.

## Materials and Methods

This was a cross-sectional study, and data on knowledge, perceptions, and selection of the current contraceptive method were collected.

In this study, “knowledge” is defined as the awareness and recognition of the existence of the listed contraceptive methods. This encompasses an understanding that these methods are available options, irrespective of whether the information was acquired through personal experience, education, or other means. It does not imply in-depth understanding of usage, effectiveness, side effects, or specific attributes of the methods.

### Participants

Participants were recruited from the IQVIA panel, where registered women received invitations to participate. The term “women” was defined as individuals assigned female at birth who identify as female, with inclusion criteria being residence in Brazil and aged 18–49 years. Exclusion criteria included women who do not menstruate, were in menopause, or have undergone hysterectomy, salpingectomy, and/or oophorectomy. The recruitment aimed for a sample reflective of Brazil’s demographic distribution in terms of region, as outlined by the 2010 Brazilian Census, using a quota sampling method to ensure proportional representation across Brazil's regions (Southeast, Northeast, South, North, and Central-West), and also in terms of socioeconomic classes (A, B, C, D-E).^9^ (see Supplementary Data S1).

The sample size of 2000 was calculated to have a maximum error of ± 2.2% at a 95% confidence interval (CI). The survey specifically targeted women who were currently using a contraceptive method. An additional 1000 responders who were not using any contraceptive method answered sociodemographic questions and the reason for not using any method.

The socioeconomic classification was based on purchasing power and defines five classes from A to E, in which A is the highest purchasing power and E is the lowest, according to *Critério de Classificação Econômica Brasil*.^10^

All participants signed an informed consent form, and this study was approved by the Ethics Committee of Invitare Clinical Research, CAAE 55677422.1.0000.8098.

### Questionnaire/survey

Data were collected from March to July 2022 through an online survey, and all responses were anonymized. The questionnaire is available in the Supplementary Material.

### Statistical analysis

Analysis was conducted using the SAS software version 9.4. Continuous variables were summarized using descriptive statistics, whereas qualitative variables were summarized using absolute and relative frequencies (%).

The impact of age, socioeconomic class, and region, as well as possible interactions between these factors, was evaluated using multivariate logistic regression or chi square tests. Results were considered significant if *p* < 0.05, with a 95% CI.

## Results

Age distribution in the total sample (3000) was 27.8% aged 18–25 years, 33.4% aged 26–35 years, and 38.9% aged 36–49 years. Henceforth, the data presented are from the target population, *i.e.*, the 2000 participants using a contraceptive method, and data about the 1000 women not using any contraceptive method are presented at the end. The following table outlines sociodemographic data for the target population (Table 1).

**Table 1.** Distribution of Participants (*n* = 2,000) by Age Group, Socioeconomic Class, and Region, Represents Percentages Aligned with the 2010 Sociodemographic Data of the Brazilian Population

	Socioeconomic class				
Age/region	A (upper)		B (Mid-up)		C (mid-low)	D–E (lower)	Total
Southeast (n; %)					
Age (years)					
18–25	7 (0.4%)		54 (2.7%)		92 (4.6%)	80 (4,0%)	233 (11.7%)
26–35	18 (0.9%)		82 (4.1%)		124 (6.2%)	54 (2.7%)	278 (13.9%)
36–49	9 (0.5%)		130 (6.5%)		150 (7.5%)	20 (1.0%)	309 (15.5%)
Southeast Total	**34 (1.7%)**		**266 (13.3%)**		**366 (18.3%)**	**154 (7.7%)**	**820 (41.0%)**
Northeast (n; %)							
Age (years)							
18–25	2 (0.1%)		3 (0.2%)		51 (2.6%)	95 (4.8%)	151 (7.6%)
26–35	8 (0.4%)		8 (0.4%)		69 (3.5%)	69 (3.5%)	154 (7.7%)
36–49	6 (0.3%)		6 (0.3%)		196 (9.8%)	47 (2.4%)	255 (12.8%)
Northeast Total	**16 (0.8%)**		**17 (0.9%)**		**316 (15.8%)**	**211 (10.6%)**	**560 (28.0%)**
South (n; %)							
Age (years)							
18–25	1 (0.1%)		16 (0.8%)		28 (1.4%)	30 (1.5%)	75 (3.8%)
26–35	4 (0.2%)		31 (1.6%)		36 (1.8%)	26 (1.3%)	97 (4.9%)
36–49	5 (0.3%)		38 (1.9%)		45 (2.3%)	20 (1.0%)	108 (5.4%)
South Total	**10 (0.5%)**		**85 (4.3%)**		**109 (5.5%)**	**76 (3.8%)**	**280 (14.0%)**
North (n; %)							
Age (years)							
18–25	0 (0.0%)		4 (0.2%)		21 (1.1%)	26 (1.3%)	51 (2.6%)
26–35	0 (0.0%)		3 (0.2%)		34 (1.7%)	17 (0.9%)	54 (2.7%)
36–49	0 (0.0%)		30 (1.5%)		38 (1.9%)	7 (0.4%)	75 (3.8%)
Noth Total	**0 (0.0%)**		**37 (1.9%)**		**93 (4.7%)**	**50 (2.5%)**	**180 (9.0%)**
Central-West (n; %)							
Age (years)							
18–25	0 (0.0%)		10 (0.5%)		14 (0.7%)	17 (0.9%)	41 (2.1%)
26–35	0 (0.0%)		20 (1.0%)		32 (1.6%)	8 (0.4%)	60 (3.0%)
36–49	0 (0.0%)		25 (1.3%)		30 (1.5%)	4 (0.2%)	59 (3.0%)
Central-West total	**0 (0.0%)**		**55 (2.8%)**		**76 (3.8%)**	**29 (1.5%)**	**160 (8.0%)**
Overall total	**60 (3.0%)**		**460 (23.0%)**		**960 (48.0%)**	**520 (26.0%)**	**2000 (100.0%)**

Many respondents were married/civil union (53.3%), 39.6% were single, 5% were divorced, and 0.3% were widowed or had other status (1.9%). Most participants were parents (68.8%), and of these, most had 1–2 children (80.4%). Nearly all participants (94.3%) reported being sexually active, defined as having had a sexual relation within the past year. Most participants had 12 or more years of education (86.5%), including 28.4% with a college degree and 7.7% with a postgraduate degree. Only 13.7% of the participants had less than 12 years of education, 6.8% had 9–11 years, 4.3% had 5–8 years, and 2.6% had <5 years).

Among the participants, 67.3% (1320) were economically active, and 34.2% of them were largely responsible for their household income, accounting for 45%–60% of the family income.

### Knowledge about contraceptive methods

Based on the responses of the target population, the three most known methods were oral contraceptives (87.3%), external condoms (63.6%), and the copper IUD (45.1%).

Women aged 18–25 years reported significantly greater knowledge than other age groups for the following methods: copper and hormonal IUDs, contraceptive injections, morning-after pills, contraceptive implants, and the cervical mucus method. Women aged 36–49 years were more aware of the calendar-based method than others (Table 2).

**Table 2.** Percentage Distribution of Participants Who Reported Knowing the Presented Contraceptive Methods, by Age

	Age group				
Contraceptive methods-(%)	18 −25 years (*n* = 551)	26–35 years (*n* = 643)	36–49 years (*n* = 806)	Total (*n* = 2,000)	*p*-value^a^
Oral contraceptive/oral daily pill	88.2%	86.0%	87.5%	87.3%	*p* = 0.501
External condom	61.2%	62.1%	66.3%	63.6%	*p* = 0.100
Copper IUD	**50.6%** ^b^	44.3%	**41.6%** ^b^	45.1%	***p* = 0.004**
Contraceptive injection	**45.0%** ^b^	35.3%	**34.4%** ^b^	37.7%	***p* < 0.001**
Hormonal IUD	**40.7%** ^b^	35.5%	**33.5%** ^b^	36.2%	***p* = 0.023**
Internal condom	**39.6%** ^b^	**32.7%** ^b^	36.3%	36.1%	***p* = 0.045**
Emergency contraceptive	**35.4%** ^b^	27.1%	**22.8%** ^b^	27.7%	***p* < 0.001**
Tubal ligation	21.2%	19.4%	21.4%	20.8%	*p* = 0.612
Contraceptive implants	**25.0%** ^b^	20.1%	**13.0%** ^b^	18.6%	***p* < 0.001**
Contraceptive skin patches	**18.0%** ^b^	12.8%	**11.8%** ^b^	13.8%	***p* = 0.003**
Partners vasectomy	11.8%	10.7%	12.1%	11.6%	*p* = 0.695
Calendar-based/rhythm method	**7.8%** ^b^	8.9%	**13.8%** ^b^	10.5%	***p* < 0.001**
Coitus interruptus/withdrawal	8.5%	7.6%	9.2%	8.5%	*p* = 0.575
IUD (unspecified)	**3.4%** ^b^	6.2%	**9.5%** ^b^	6.8%	***p* < 0.001**
Vaginal ring	5.1%	4.8%	3.0%	4.2%	*p* = 0.094
Diaphragm/sponges	2.9%	3.7%	3.7%	3.5%	*p* = 0.672
Spermicide use/spermicide lotion	1.5%	2.0%	1.6%%	1.7%	*p* = 0.726
Cervical mucus/Billings method	**1.8%** ^b^	1.1%	**0.4%** ^b^	1.0%	***p* = 0.031**
Basal temperature method	0.7%	0.5%	0.2%	0.5%	—

The participants could choose more than one answer (question with multiple choice alternatives).

^a^ Chi square test.

^b^ Statistic difference between groups.

IUD, intrauterine device.

When compared with middle and upper classes, the D–E socioeconomic classes exhibited less knowledge (*p* < 0.05) regarding calendar-based methods (8.3%), contraceptive patches (10.9%), hormonal (30.3%) and copper (38.8%) IUDs, and external condoms (57.6%). Classes A and B had a greater (53%) chance of knowing about copper IUDs than classes D–E. However, classes D–E had more knowledge about contraceptive injection (44.0%) and tubal ligation (25.1%).

Regional variations in knowledge of contraceptive methods were found. Women in Central-West demonstrated significantly better knowledge (*p* < 0.05) of hormonal IUDs (48.1%) and contraceptive patches (20%), whereas the Northeast was less aware of this method (10.5%).

### Current use of contraceptives

Contraceptive pills, external condoms, contraceptive injections, and copper and hormonal IUDs were the five most frequently used methods in Brazil.

**FIG. 1.**
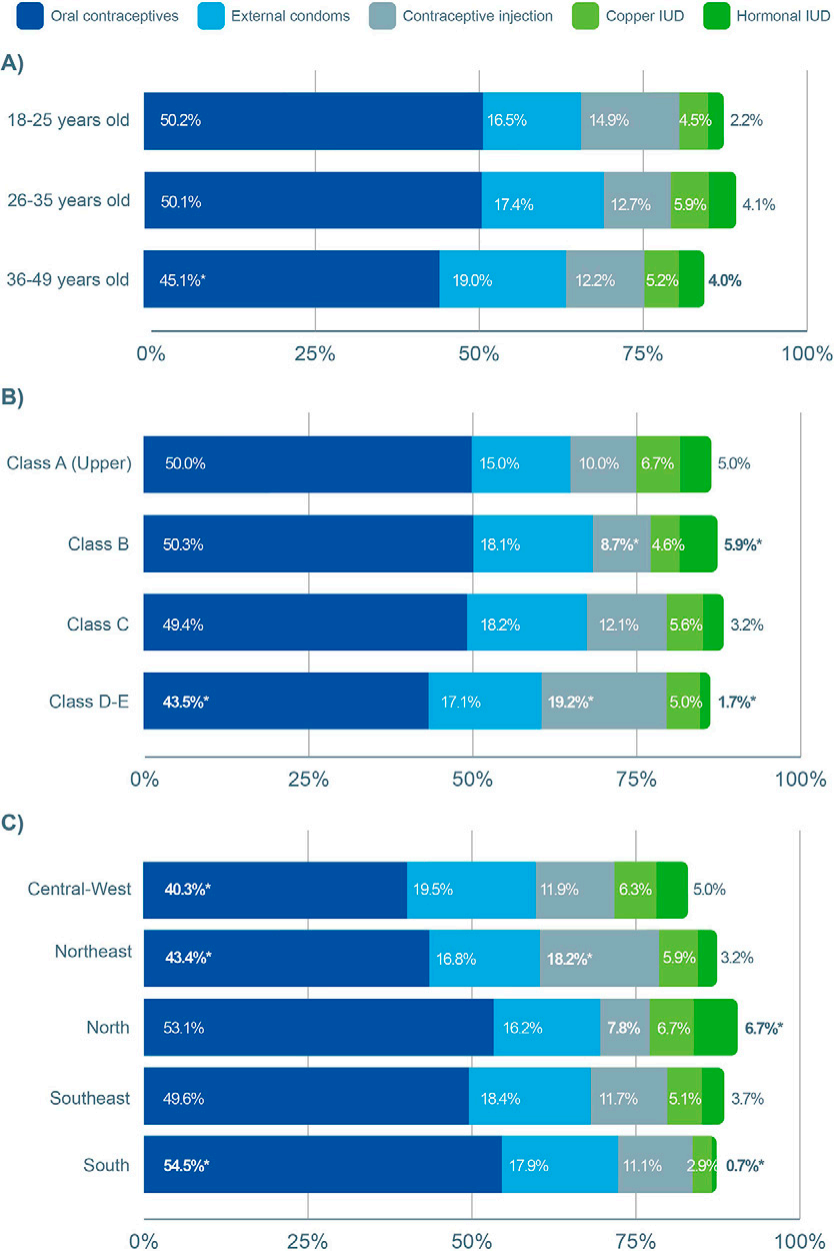
Contraceptive use of the five most known methods stratified by age, socioeconomic class, and region: **(A)** By age groups, **(B)** By socioeconomic class, and **(C)** By Brazil region. Statistical significance is denoted as follows: (*p* > 0.05) for age groups, (*p* < 0.001) for socioeconomic class, and (*p* < 0.05) for Brazil regions. IUD, intrauterine device. Asterisks (*) indicate statistical differences between groups: when a single value is indicated, it is significantly different from the other two; when two values are indicated, they are different from each other; when three values are indicated, the three are different from each other), as determined by chi-square tests.

There was no difference in the prevalence of oral contraceptive use by age. The use of oral contraceptives was lower among the lowest income classes (D–E) (43.5%), while contraceptive injections were more frequently used (19.2%) compared with class B (8.7%) (*p* < 0.001). Age had no impact on the use of contraceptive injections. Hormonal IUDs were more commonly used by class B (5.9%), as opposed to classes D–E (1.7%) (*p* < 0.001). When analyzing regional data, oral contraceptives were less used in the Central-West (40.3%) and Northeast (43.4%) and more used in the South (54.5%) (*p* = 0.014). Contraceptive injections were more used in the Northeast (18.2%) (*p* = 0.014). In addition, hormonal IUDs were more frequently used in the North (6.7%) than in the South (0.7%) (*p* = 0.014), data presented in Figure 1.

The use of external condoms and copper IUDs was not significantly related to age, socioeconomic status, or region. Further results of the multivariate analysis on contraceptive method usage are presented in the Supplementary Material.

### Reasons to use contraception

All answers to “Reasons to use contraception” by age are presented in Table 3. Younger women reported “Prevent pregnancy” significantly more (*p* < 0.001). In addition, an age effect was observed, with declining mentions of “menstrual flow control” and “menstrual cycle regulation” as age increases (*p* < 0.001). “Hormonal control” was more frequent in the 26–35 age group and less frequent in the 36–49 age group (*p* = 0.049); “improving the skin” was less frequent in the 36–49 age group (*p* = 0.002), and “it’s the same as what a family member/friend uses,” was more frequent in the 18–25 age group (*p* < 0.001).

**Table 3.** Reasons to Use Contraception by Age Groups (Participants Could Select Multiple Answers)

	Age group							
Reasons-n (%)	18–25 years (*n* = 551)		26–35 years (*n* = 643)		36–49 years (*n* = 806)		Total (*n* = 2000)	*p*-value^a^
Prevent pregnancy^b^	489	95.5%^c^	569	93.4%	687	89.8%	1745 (92.5%)	*p* < 0.001^c^
Control menstrual flow	**202**	**36.7%^c^**	**192**	**29.9%^c^**	**197**	**24.4%^c^**	591 (29.6%)	***p* < 0.001^c^**
Regulate menstrual cycle	**173**	**31.4%^c^**	**160**	**24.9%^c^**	**150**	**18.6%^c^**	483 (24.2%)	***p* < 0.001^c^**
Protection against STIs	91	16.5%	86	13.4%	102	12.7%	279 (14.0%)	*p* = 0.115
Hormonal control	70	12.7%	**85**	**13.2%^c^**	**76**	**9.4%^c^**	231 (11.6%)	***p* = 0.049^c^**
Improve PMS symptoms	68	12.3%	56	8.7%	79	9.8%	203 (10.2%)	*p* = 0.107
Improve skin/acne	61	11.1%	58	9.0%	**47**	**5.8%^c^**	166 (8.3%)	***p* = 0.002^c^**
Because of a gynecological disease**^d^**	17	3.1%	24	3.7%	29	3.6%	70 (3.5%)	*p* = 0.816
It is the same as a relative/friend	**27**	**4.9%^c^**	16	2.5%	11	1.4%	54 (2.7%)	***p* < 0.001^c^**
Others	10	1.8%	21	3.3%	26	3.2%	57 (2.9%)	*p* = 0.229

^a^ Chi-square test.

^b^ Possible answer was only available for the 94.7% (1886) participants who were sexually active.

^c^ Represent those with statistical differences between groups (when a single value is indicated, it is significantly different from the other two; when two values are indicated, they are different from each other; when three values are indicated, the three are different from each other).

^d^ Most cited gynecological diseases were polycystic ovaries (37.1%), uterine myoma (18.6%), and endometriosis (17.1%).

PMS, premenstrual syndrome.

Middle-up class women (35.2%) presented a higher prevalence of “menstrual flow control,” whereas higher-income women (36.7%) reported a higher prevalence of “menstrual cycle regulation,” with lower-income women (18.8%) showing less prevalence.

“Hormonal control” was more prevalent in Class B (15.0%) and less so in D-E (7.7%); “Improving symptoms such as PMS” was more prevalent in Class B (13.2%) and less so in D-E (5.8%). “Improving the skin” (16.7%) and “it is the same that a family member/friend uses” (8.3%) were significantly more reported in social class A, and “due to some gynecological disease,” was less reported in social class B.

Moreover, Southern women reported “prevent pregnancy” more than those in the Northeast (95.5% versus 90.1%). However, “other reasons” for choosing the contraceptive method were not different between regions of the country.

### Satisfaction with current contraceptive method

**FIG. 2.**
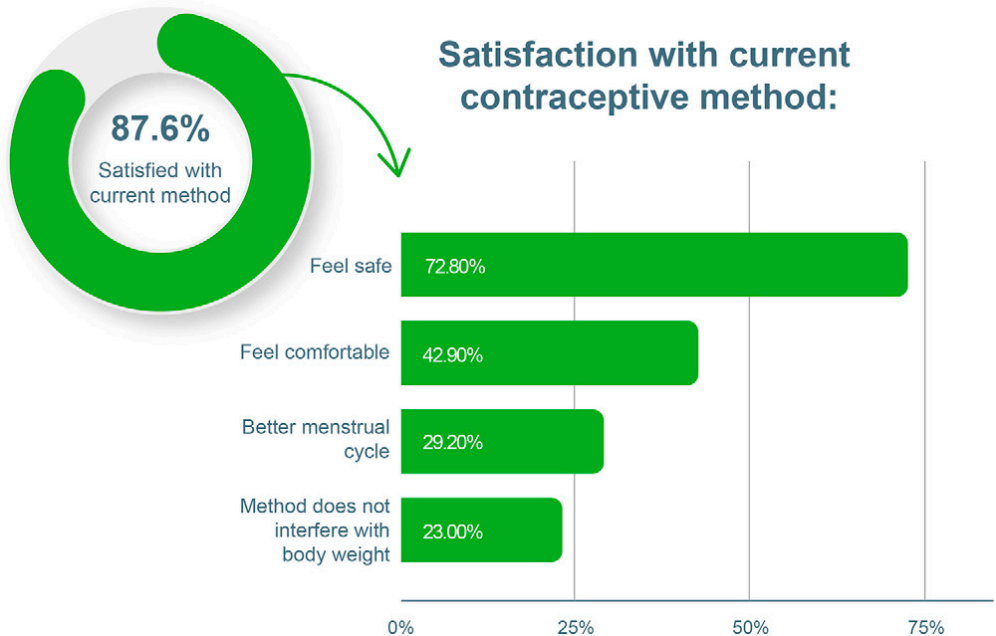
Descriptive data illustrating satisfaction levels with current contraceptive methods and the main reasons cited for satisfaction among participants. Participants had the option to select multiple responses, as the question provided multiple-choice alternatives.

Nearly all women (87.6%) were satisfied with their current method, and the most mentioned were: contraceptive pill (47.2%), male condom (17.8%), hormonal injection (13.3%), copper IUD (5.5%), and hormonal IUD (3.8%). The main reasons for satisfaction included “feel safe” (72.8%), “feel comfortable” (42.9%), “better menstrual cycle (29.2%), and “Method does not interfere with body weight” (23%) (Fig. 2).

The main reasons cited by respondents who were not satisfied with their current method were “it was too expensive” (31.7%), the “physician had not yet recommended it” (22.5%), “no free access through the public health system” (16.9%), and “not knowing how to acquire the desired method” (14.5%). Of the participants who reported dissatisfaction, the most frequent method considered ideal was the copper IUD (18.1%), followed by the partner's vasectomy (17.3%), hormonal IUD (14.5%), and tubal ligation (14.1%).

### Reasons for switching and discontinuing contraceptives

**FIG. 3.**
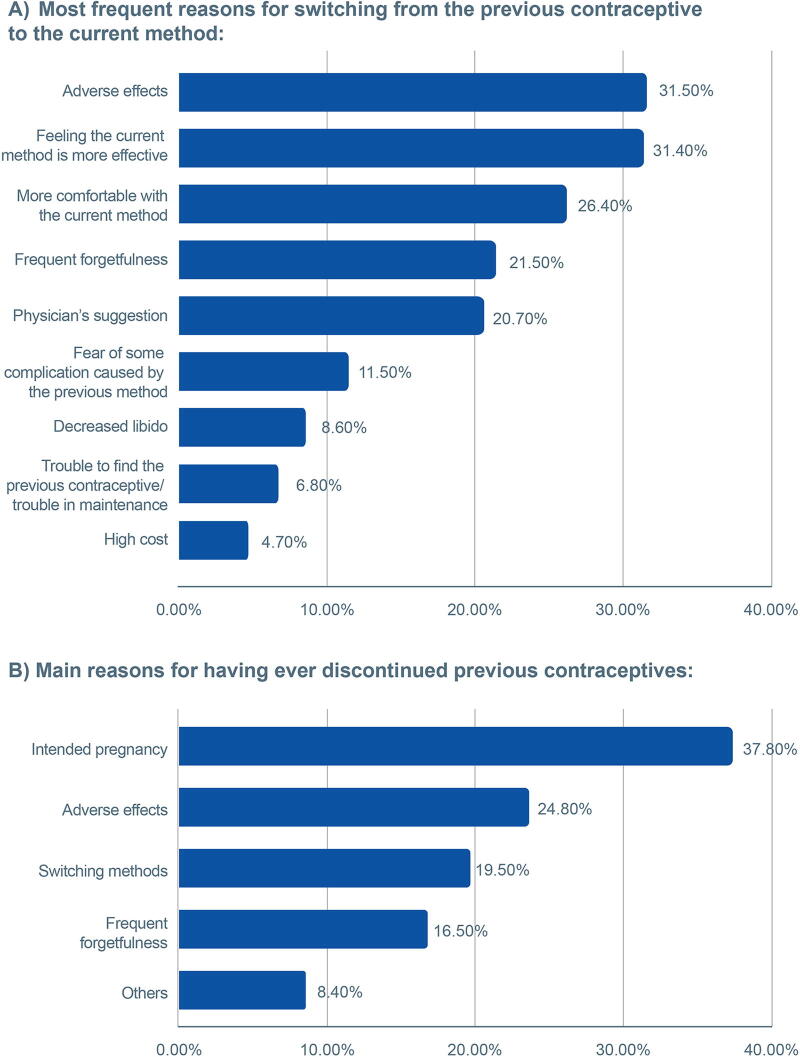
Participant responses to multiple-choice questions, where more than one option could be selected. **(A)** Reasons for switching from previous contraceptive methods to current ones. **(B)** Reasons for discontinuing the use of previous contraceptives.

Among the participants who currently use contraceptives, 70.1% said they had already used another method. The most frequent reasons for switching from a previous method to the current one were adverse effects (31.5%), feeling the current method is more effective (31.4%), and feeling more comfortable with the current method (26.4%). For discontinuing contraceptive use, the main reasons cited were intended pregnancy (37.8%), adverse effects (24.8%), and switching methods (19.5%) (Fig. 3).

Participants aged 18–25 years showed a significantly lower likelihood of pausing the current method of contraception because of intended pregnancy (18.9%) and a higher chance of discontinuing because of forgetfulness (25.7%) and lack of financial resources (17.9%) (*p* < 0.05).

### Who or what influenced your decision about the current method?

Physicians had the greatest influence (53.9%) over the decision to use the current method, followed by “no one influenced my choice” (20.1%), family members (16.8%), partners (13.8%), and other healthcare providers (13%). Friends (8.4%), internet searches (7.6%), talks in school/college (6.6%), advertisements (3.5%), teachers (2%), and social networks (0.7%) were less cited as influences. The majority of physicians who influenced the choice were gynecologists/obstetricians (90%) and 9.3% were general practitioners.

Age had a significant effect when evaluating multivariate factors (*p* < 0.05), as only 13.8% of women in the 18–25 age group said, “No one influenced my choice,” and this percentage increases with age. Younger women were more externally influenced, particularly by family, partners, friends, internet searchers, school, and teachers. Social classes A (71.7%) and B (61.3%) were more influenced by the “Physician” than mid-low-class C (55.4%) and lower strata (42.5%). Socioeconomic class B (11.5%) was more influenced by “internet searches,” whereas class C (5.8%) was less influenced by it. The prevalence of “No one influenced my choice” was higher in the Northeast (24.5%) than in other regions.

### The choice of the current contraceptive was mainly based on which factor?

**FIG. 4.**
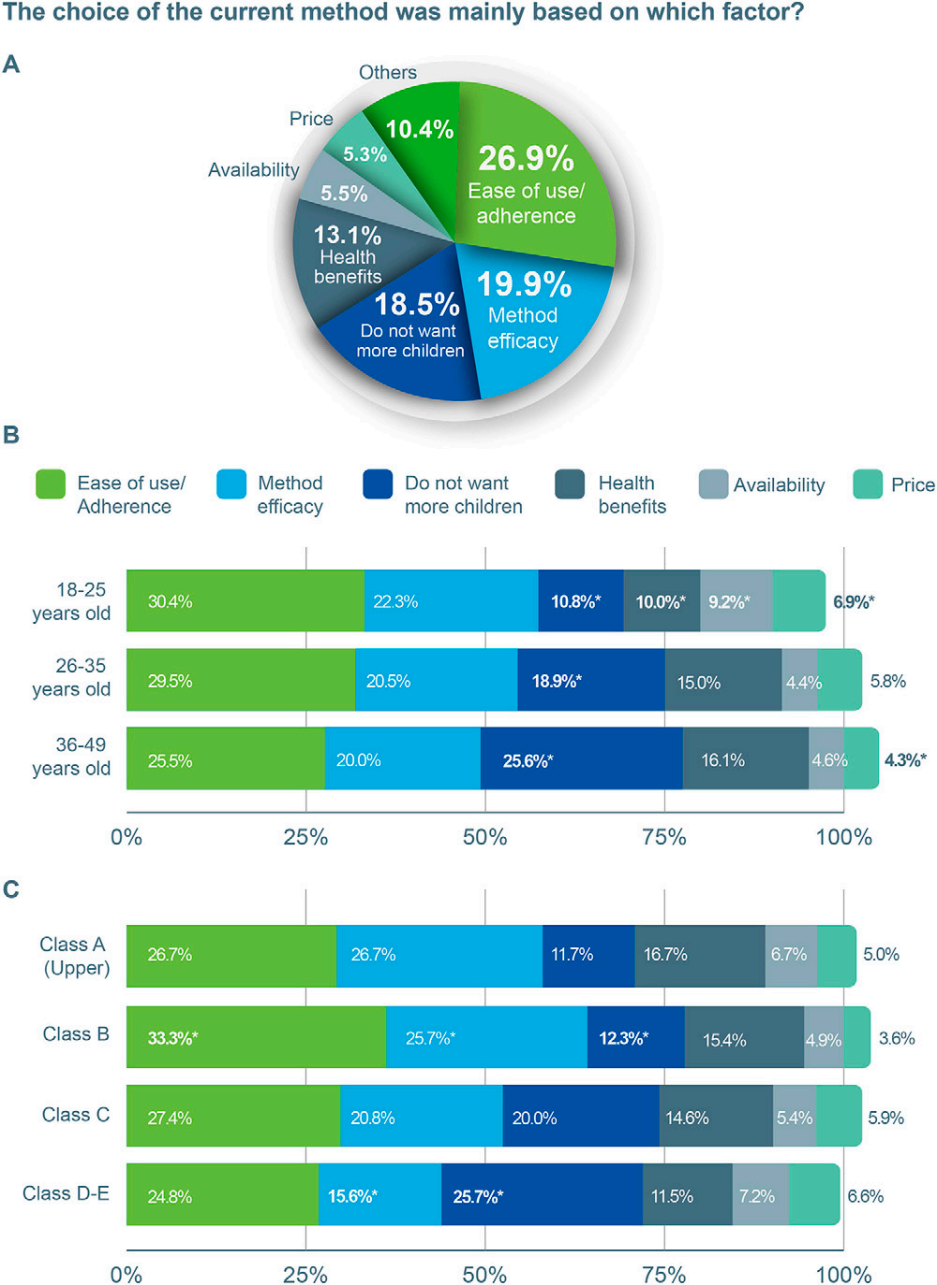
Overview of participants’ choices regarding their current contraceptive method. Participants could only choose one answer to this question. **(A)** Main reasons cited for choosing the current contraceptive method. **(B)** Main reasons by age group. **(C)** Main reasons, by socioeconomic class. Asterisks (*) denote statistical significance (*p* < 0.001) as determined by chi-square tests: a single asterisk indicates a significant difference from all other groups, two asterisks denote differences between two groups, and three asterisks signify differences among three distinct groups.

Most (26.9%) participants cited “Ease of use/adherence” as the primary reason, whereas 19.9% chose “method efficacy” and 18.5% chose the method specifically because they “do not want more children” (Fig. 4A). Methods with the highest association to “Ease of use/adherence” were the contraceptive pill (28.7%), followed by the male condom (14%), and the hormone injection (12.8%).

The factor “I don't want to have more children” was less mentioned in the 18–25 age group (10.8%) and more in older age groups (*p* < 0.001, Fig. 4B). In younger women, “Health benefits” was less mentioned (10.0%), whereas “product availability” (9.2%) was mentioned more (*p* < 0.001), and “price” was mentioned more (6.9%, *p* < 0.001).

Class B cited “ease of use” more than other classes. “Method efficacy” was significantly more prevalent in class B (25.7%) and less in classes D–E (15.6%), whereas “I don’t want to have more children” was less cited by the middle-up class B (12.3%) and more by lower classes D–E (25.7%) (*p* < 0.001, Fig. 4C). For the lower-income classes D–E, “method efficacy” was less cited (15.6%) (*p* < 0.001). No variations were observed across Brazilian regions.

### Who pays for your contraceptive method?

Most respondents (63.1%) paid for their own contraceptives. Sexual partners (24.7%) and family (2.4%) were other frequently mentioned payers. Public healthcare systems (SUS) facilitated contraceptive access for 18.1% of women. Younger women were more likely to have their family as the payer (5.9%). Most women in higher economic classes paid for their contraceptives (82.8% in class A and 72.8% in class B). This percentage dropped for classes D–E to 57.7%, and those from lower-income classes accessed contraception *via* SUS more often (27.0%), compared with those in class B (7.1%) and class A (6.9%). Access through SUS was more significant in the Northeast (23.3%) and less in the Southeast (15.3%).

### Reasons for not using a contraceptive method

Of the 1000 participants who were not using any contraceptive method, the main reasons were “Do not have a current partner” (26.9%) and “planned or current pregnancy” (17.1%), other reasons are presented in Table 4.

**Table 4.** The Reason Why the 1000 Women Enrolled in This Study Decided Not to Use a Contraceptive Method

Reasons	n (%)
Do not have a current partner	269 (26.9%)
Planned or current pregnancy	171 (17.1%)
Not able to get pregnant	106 (11.6%)
Not knowing the best method	80 (8%)
In a relationship with a woman	53 (5.3%)
The desired method is too expensive	42 (4.2%)
Health restriction^a^	33 (3.3%)
Others	226 (22.6%)

^a^ The most alleged health restrictions were thrombosis, “allergy to oral contraceptives” and cardiac alteration.

## Discussion

Our study provides valuable insights into current contraceptive knowledge and use in Brazil, considering the influence of sociodemographic factors. Moreover, our analysis further explores women’s contraceptive choices, satisfaction, and who or what influences these choices.

Regarding distinct levels of contraceptive knowledge, younger women knew more about modern methods: contraceptive pills, condoms (external and internal), IUDs, sterilization (male and female), injectables, diaphragms, spermicides, and emergency contraception, while more mature women knew more about natural methods, possibly a cultural effect and a result of increased sexual education and information access. Socioeconomic classes also influenced knowledge, with lower-income groups (D–E) less aware of several modern methods, such as external condoms and IUDs, compared with their higher-income counterparts. On the contrary, they had more familiarity with options like contraceptive injections and tubal ligation. Understanding these patterns is crucial since knowledge significantly impacts the adoption of contraceptive methods. While tubal ligation is an inexpensive, permanent solution, increased awareness can guide women toward other effective, yet reversible, contraceptive options, allowing them to make informed choices aligned with their family planning goals and preferences.

Our findings reveal a notable shift in contraceptive preferences in Brazil, aligning with national trends. The National Health Survey (NHS) in 2013 reported that 25.9% of women used surgical methods and 34.2% used oral contraceptives.^6^ By NHS-2019, these numbers shifted to 17.3% for tubal ligation and 40.6% for oral contraceptives.^11^ Our study further underscores this trend, showing tubal ligation has dropped to less than 15% and an even higher prevalence of oral contraceptive use, at 48.1%. Furthermore, tubal ligation was more cited by older women (5.6%), which reflects a growing inclination toward reversible contraception and demonstrates enhanced awareness and decision making among Brazilian women regarding their contraceptive choices. Moreover, the higher education level prevalent in our sample, typically associated with higher socioeconomic status, might also explain a higher adoption of oral contraceptives.

Besides, oral contraceptives are more used by young adults, and the use decreases with age. Previous authors had hypothesized that the youth preference for oral contraceptives was because of the noncontraceptive benefits of the pills, *e.g.*, more favorable bleeding patterns, improvement in menstruation-related symptoms, and skin improvement.^12^ Our study supports this hypothesis as the most frequently cited noncontraceptive reasons for using the current method were “to control” and “to regulate” menstrual flow, being more frequent among younger women and those in higher-income classes.

In addiiton, we observed a trend toward decreased use of oral and increased use of injectable contraceptives among the lowest income classes. Compared with contraceptive pills, injectables have higher efficacy in common-use scenarios, with a failure rate of 6% compared with 9% for pills and lower maintenance requirements.^13–15^ However, contraceptive injections have come under scrutiny in the first decades of use because of safety concerns and have been shown to lead to weight gain, obesity-related comorbidities, and delayed return to fertility.^15,16^ Injectables are more frequently used by marginalized populations and those with less access; for example, 87.9% of women aged 16–56 years who used contraceptive injections in the United States were African Americans,^14^ and in New Zealand, most women who used contraceptive injections were Māori, from low-income or lower education levels.^17^ This may be because of a public health strategy or women’s choice to go for nondaily methods, while it could also represent reduced healthcare access. Other long-acting reversible contraceptive options are copper and hormonal IUDs. In our study, while copper IUDs seem to be equally used independently of sociodemographic factors, hormonal IUDs were more prevalent among higher-income classes and rare among lower-income women, probably because of their higher costs.

Despite an overall improvement in healthcare access, Brazil still exhibits profound geographic inequalities, as the South and Southeast regions offer better access to health services.^18^ We found decreased oral contraceptive use in Central-West and Northeast, whereas other studies have detected decreased use of contraceptive pills in the North.^6,19^ Variation in the prevalence of contraceptive methods across regions may be attributed to the greater knowledge of hormonal IUDs and contraceptive patches in the Central West, indicating a preference for nondaily methods. Conversely, contraceptive injections were more frequently used in the Northeast region. Studies evaluating the regional perception and use of contraceptive methods among adult women are limited. However, one study examining sexual health indicators among school-age adolescents who have initiated sexual activity found that those in the lower-income regions of the North, Northwest, and Central-West had poorer indicators.^20^

Heterosexual intercourse remains the primary HIV transmission route for women in Brazil.^21^ Alarmingly, our findings reveal that only 17.8% of participants reported using external condoms, despite just over half (53.1%) being married. Moreover, a mere 14.0% of interviewees cited STI prevention as a reason for condom use. This underrepresentation could be attributed to our survey's emphasis on contraceptives, potentially underestimating condom use for noncontraceptive purposes. In addition, our data did not show a significant association between condom use and sociodemographic factors but highlighted that lower-income classes possess less knowledge about external condoms. These results underscore critical concerns in sexual health education and align with broader trends observed in Brazil such as the increase in the rate of acquired syphilis and cases of HIV infection.^22,23^ For instance, a 2019 study, with 88,531 Brazilians over 18 years of age, found that only 20.9% of Brazilian women reported consistent condom use over the past year.^20^ Similarly, the NHS-2019 showed that merely 20.4% of women used condoms as a contraceptive method.^11^ These findings suggest a need for future research to delve deeper into the factors influencing condom use among Brazilian women, beyond just contraception.

One key finding of this present study is regarding the reasoning behind contraceptive choice. Herein, the main reason was “ease of use/adherence,” and the leading reasons for satisfaction were related to safety (adverse effects, seeking to feel more secure, or comfortable) and adherence (frequent forgetfulness on the previous method). Diving into the main reason for choosing the current method, compared with other ages, younger women considered price and product availability more and health benefits less. Lower-income women, compared with the middle-up class, were more driven by “do not want more children” and less by “method efficacy,” possibly because poorer women tend to have more children (the national mean of children/women is 1.7, but 2.9 for poorer women).^24^ Physicians tend to prefer high-efficacy methods, and as they are the most mentioned influencers on method choice, they should be aware of their patients’ preferences and reasons for use.^25^

Lower-income strata presented less physician influence, possibly reflecting diminished access to gynecological care. Younger women are more influenced by others like family, partners, friends, internet searches, teachers, and advertisements. Data from Brazilian teenagers have also shown them to be more influenced by family and friends on contraceptive choice.^26^ Advertisement and internet content must be regulated to provide helpful and true information, while healthcare providers should also make an effort to comprehend young women’s needs.

All available contraceptive methods have pros and cons. With this consideration, patient centricity is a core component of high-quality healthcare, which involves helping patients choose a method that meets their needs and preferences.^8^ If the woman feels safe and comfortable, this will likely increase satisfaction and adherence, as the most frequent reasons for switching were related to adherence and safety. Adverse effects were also an important reason for discontinuing the use of contraceptives, second only to “intended pregnancy,” although younger respondents (18–25 years) were more likely to stop because of forgetfulness (25.7%) and lack of money (17.9%). Dissatisfaction was associated with cost and difficulty of access. Most (63.1%) participants paid themselves for the contraceptive, which is not surprising, as most were economically active. Younger and poorer women were more dependent on others or the healthcare system.

Contraceptive methods offered at no cost by the public healthcare system in Brazil are oral contraceptive pills, copper IUDs, injectables, external and internal condoms, diaphragm, emergency contraceptives, tubal ligation, and vasectomy. For highly vulnerable populations (homeless people, on pre-exposure prophylaxis, and sex workers), hormonal IUDs and contraceptive implants can be considered. Except for natural methods, condoms, and spermicide, other contraceptive methods are only available by prescription.^15^ Thus, contraceptive methods are associated with healthcare access.^1^ Furthermore, 16.9% of respondents who were not satisfied with their current contraceptive, declared they had not changed because the ideal method was not available for free.

Of the women who were not using contraceptives, 8% were because of being unaware of the best method, 4.2% because of lack of access to the desired method, and 3.3% because of health restrictions. Added, this shows that 15.5% of women not using contraceptives had an unmet need for gynecological health. According to the United Nations, about 10% of women of reproductive age have an unmet need for family planning.^27^

Our study offers a significant contribution to understanding contraceptive use among Brazilian women, with its major strength being the inclusion of participants from all regions and socioeconomic classes. However, the study’s reliance on an online survey excludes the 20.9% of the population without internet access.^28^ In addition, the overrepresentation of higher educated participants and the self-reported data could introduce biases. While the quota sampling addresses regional and socioeconomic diversity, it does not capture other influential factors like cultural or racial diversity and urban–rural distinctions. Despite these limitations, the study’s broad scope and focus on a vital public health topic offer valuable insights, albeit with caution because of its cross-sectional nature and potential biases.

Modern contraception enables women to choose when and how to give birth, but dissatisfaction, misuse, and inequalities remain. Although effectiveness is crucial, providers must consider other important factors for each patient. Physicians should evaluate individual preferences, access, and previous experience to recommend a method that is likely to result in patient satisfaction and adherence.

## Conclusion

Our findings demonstrate that younger generations have better knowledge of contraceptive options. Modern contraceptives, especially oral pills, external condoms, contraceptive injections, and copper IUDs, emerged as the most used and recognized methods. We observed a significant shift toward reversible methods, particularly oral contraceptives, with tubal ligation rates declining. Our results highlight the impact of socioeconomic factors on contraceptive choices, suggesting a need for increased public awareness campaigns and improved access policies, especially for lower-income groups. Addressing regional disparities and promoting informed decision making will be crucial in shaping effective reproductive health policies and interventions in Brazil.

## Supplementary Material

Supplementary Data S1

## Abbreviations Used

CI: confidence interval
HIV: human immunodeficiency virus
IUD: intrauterine devices
NHS: National Health Survey
PMS: premenstrual syndrome
STI: sexually Transmitted Infections
SUS: Public healthcare systems
