# Supplementary material for: Knowledge, Use, and Perception of Brazilian Women about Contraceptive Methods: An Observational Study
Source: Womens Health Rep (New Rochelle). 2024 May 27;5(1):460–72. doi: 10.1089/whr.2023.0185 (PMC11257120; doi:10.1089/whr.2023.0185)
Supplement: Supplementary Data S1 [file whr.2023.0185_supp_datas1.docx]

**SUPPLEMENTARY MATERIAL**

**Knowledge, use, and perception of Brazilian women about contraceptive methods: An Observational Study**

Juliana Dineia Perez Brandão^a*^; Rogerio Bonassi Machado^b^, Ana Carolina Ferreira Cardoso^a^.

^a^ Libbs Farmacêutica Ltda, Scientific Medical Division, São Paulo,

Address: Av. Marques de São Vicente, 2219 – 2 floor – Jardim das Perdizes

zip code 05036-040 São Paulo – São Paulo, Brazil

^b^ Department of Gynecology and Obstetrics, Jundiai School of Medicine, Jundiai, Brazil.

*Correspondent author

**Index**

[**1. Methodology: Sampling Strategy and Representativeness Details 2**](#_6advrwbaiv9y)

[1.1. Sample Recruitment and Proportional Representativity 2](#_pkj6aa5rnyqo)

[1.2. Data Collection and Limitations 3](#_wag8cuwv7yii)

[1.3. Analysis 3](#_isbdu2g6nhiw)

[**2. Results 4**](#_lde12hyzep2n)

[2.1. Brazilian HDI per region and the six most used methods per region. 4](#_tytjdvmi57a)

[2.2. Odds ratio from analysis of chances of use for different methods 5](#_sc28o140jefm)

[2.2.1. Oral contraceptive pills 5](#_fqh18hdjgq12)

[2.2.2. Contraceptive Injections 6](#_t6vsicxiefb)

[2.2.3. External condoms and Copper IUD 6](#_w9s87u4rxtit)

[2.3. Who was responsible for the decision on your current contraceptive method? 7](#_p7ci6re1lnbm)

[**3. References 8**](#_nq0yn7l2td5e)

[**4. Questionnaire 9**](#_z22f4nallgyr)

# **Methodology: Sampling Strategy and Representativeness Details**

## **Sample Recruitment and Proportional Representativity**

**Participants were recruited from the IQVIA panel, a diverse pool of individuals from various regions and socioeconomic backgrounds across Brazil.**

**To align our sample with the national demographic distribution, we implemented a quota sampling method. Quotas were established based on the distribution of the female population aged 20 to 49 years across Brazilian regions and socioeconomic classes, as reported in the 2010 Census [1]. The recruitment process continued until the predetermined quotas reflective of this distribution were met, ensuring that our sample mirrored the national population's geographic and socioeconomic structure.**

**We reached 2,000 responses from women aged 18-49 using contraceptive methods, aligning with national demographics, as detailed in Table S1. This stratified sampling approach ensured broad representativeness across Brazil's regions and socioeconomic classes (A, B, C, and D-E).**

**Table S1:** Comparison of Regional Distribution of Women Aged 20-49 in Brazil According to 2010 Census Data with the Regional Distribution of Survey Respondents.

|  | **Female Brazilian Population*** | | **Study participants (IQVIA responders)** | |
| --- | --- | --- | --- | --- |
| **Brazilian Region** | **n** | **%** | **n** | **%** |
| South | 6,362,222 | 14.1% | 280 | 14.0% |
| Southeast | 19,182,562 | 42.4% | 820 | 41.0% |
| Central-west | 4,042,961 | 8.9% | 160 | 8.0% |
| North | 3,478,816 | 7.7% | 180 | 9.0% |
| Northeast | 12,170,725 | 26.9% | 560 | 28.0% |
| **Total** | **45,237,285** | **100.0%** | **2,000** | **100.0%** |

**Data represents the distribution of the female population by region within the age group of 20 to 49 years, according to the Brazilian Institute of Geography and Statistics (IBGE) 2010 Census data.

## **Data Collection and Limitations**

**Data were collected through an online survey from March to July 2022, with responses anonymized to maintain participant confidentiality. Inclusion criteria specified female residents of Brazil aged 18-49, with exclusions for those without internet access, highlighting a limitation in our methodology with respect to capturing the full spectrum of the Brazilian female population.**

**While the educational distribution of participants was not a primary stratification criterion, we acknowledge that our sample exhibited a higher level of education compared to the national average. This discrepancy is recognized as a limitation of the study, and future research should consider incorporating education level as a stratification criterion to enhance representativeness.**

## **Analysis**

**The collected data were analyzed to assess the knowledge, use, and perception of contraceptive methods among Brazilian women, with subgroup analyses conducted based on age, socioeconomic status, and region. The regional and socioeconomic proportional representativity of our sample allowed for a comprehensive analysis reflective of the diverse experiences and perspectives across different segments of the Brazilian population.**

# **Results**

## **Brazilian HDI per region and the six most used methods per region.**

**Map**

**FIGURE S1 here**

**Figure S1.** Brazilian map with Human Development Index (HDI) according to each region in 2010 [2] and the six most used contraceptive methods in each region. Asterisks mark significantly different prevalences (p<0.05).

## **Odds ratio from analysis of chances of use for different methods**

Beyond the Chi-square test for assessing differences in the relative (%) use of each method according to the independent variables, we also performed multivariate logistic regressions to evaluate the effect of each independent variable on the chances of using the four most used methods (Oral contraceptive pill, external condom, contraceptive injections, and copper IUD). Herein, we present the results of those.

### **Oral contraceptive pills**

*Age*

The odds of using this method were 50% lower in women aged 36-49 years in comparison to women aged 18-25 years (OR:1.50; 95%CI:1.19-1.89; p=0.001) and 25% less than women aged 26-35 years (OR:1.25; 95%CI:1.01-1.55; p=0.037). Differences in the chi-square for age (p<0.001) were found in methods less used: tubal ligation was more common in the 36-49 years group (5.6% versus 3.5% in general); morning-after pill was more common in 18-25 years (3.5% vs 1.6%); internal condom was less common in 36-49 (0.5% vs 1.3%).

*Sociodemographic*

Socioeconomic class was not significantly associated with chances to use oral pills (p=0.051).

*Region*

There were decreased chances of using contraceptive pills in Central-West and Northeast when compared to other regions. Specifically, Central-West presented 48% lower usage of this method compared to the South (OR:0.52, 95%CI: 0.35-0.78, p = 0.001), 43% lower than the North (OR:0.57, 95%CI: 0.37-0.88, p = 0.010), and 34% lower than the Southeast (OR:0.66, 95%CI: 0.47-0.94, p = 0.020). In the Northeast region, 37% fewer women were using contraceptive pills than women from the South (OR: 0.63, 95%CI: 0.47-0.84, p = 0.002), 32% less used in North region (OR: 0.68, 95%CI: 0.48-0.95, p = 0.026), and 21% less in Southeast (OR:0.79, 95%CI: 0.63-1.00, p = 0.048).

### **Contraceptive Injections**

*Age*

There was no effect of age on the chances of using contraceptive injections (p=0.925).

*Class*

Lower-income strata women had increased odds of using contraceptive injections. Specifically, they were 40% more likely to use this method than women from class C (OR:0.60, 95%CI:0.44-0.82, p=0.001) and 47% more likely than class B (OR:0.47, 95%CI:0.3-0.72, p=0.001), although there was no difference between class D-E and class A (however, the sample size was smaller in this group). There was no effect for age (p=0.925).

*Region*

The Northeast region presented an increased chance of contraceptive injection use. Women from the Northeast region was 142% more likely to use injectables than women from the North (OR:2.42, 95%CI:1.34-4.36, p=0.004), 58% more than the South (OR:1.58, 95%CI:1.02-2.45, p=0.043) and 40% more than Southeast participants (OR:1.40, 95%CI:1.02-1.93, p=0.039).

### **External condom and Copper IUD**

The use of external condoms was not associated with age (p=0.507), class (0.927), or region (p=0.808). Additionally, the use of copper IUDs was also not significantly related to age (p=0.579), socioeconomic class (p=0.887), or region (p=0.349).

## ***Who was responsible for the decision on your current contraceptive method?***

Most (51%) women decided alone which method they would use, 23.6% decided together with their partner, and 19.9% together with their doctor. The only statistically significant difference occurred in the 18-25 age group in which the decision was taken together with the family and is more prevalent compared to the other age groups. “The decision is mine alone” (55.4%) and “The decision was made together with my family” (3.3%) were significantly more prevalent among Classes D-E, and the decision taken together with the doctor (16.7%), the least prevalent in this same social class. No region effect was observed.

Satisfaction with the current contraceptive method was independent of age, class, or region (p>0.05). When assessing satisfaction with the current method and who was the main responsible for the method choice, “The decision is mine alone” was significantly associated with positive satisfaction (p=0.031), as 89.8% of women who were satisfied were the main responsible for the method chosen.

# **References**

1. [**Instituto Brasileiro de Geografia e Estatística. (2010). *Censo demográfico 2010: resultados gerais da amostra*. Instituto Brasileiro de Geografia e Estatística - IBGE.**](http://paperpile.com/b/ew2gJe/QtY9a) [**https://books.google.at/books?id=ExuWoAEACAAJ**](https://books.google.at/books?id=ExuWoAEACAAJ)
2. **United Nations,** [***Desenvolvimento humano nas macrorregiões brasileiras*. (2016). Programa das Nações Unidas para o Desenvolvimento - PNUD Brasil.**](http://paperpile.com/b/ew2gJe/Gppv) [**https://play.google.com/store/books/details?id=FCPXswEACAAJ**](https://play.google.com/store/books/details?id=FCPXswEACAAJ)

# **Questionnaire**

**Classification filter**

**ABBREVIATIONS:** SA: Single answer MA: Multiple Answer

**Q1.** Which of the genders below do you identify with? [SA]

Female

Male [CLOSE]

Other [CLOSE]

**Q2.** How old are you? [NUMERICAL – Range: 0 to 100 – Soft Quota: 40% between 18 to 35 years]

____ Years [If < 18 or > 49 close]

**Q2a.** Have you had your first period yet? [SA]

Yes

No [CLOSE]

**Q3.** What state do you reside in? [SA]

Acre (North)

Alagoas (Northeast)

Amapá (North

Amazonas (North)

Bahia (Northeast)

Ceará (Northeast)

Distrito Federal (Central-west)

Espírito Santo (Southeast)

Goiás (Central-west)

Maranhão (Northeast)

Mato Grosso (Central-west)

Mato Grosso do Sul (Central-west)

Minas Gerais (Southeast)

Pará (North)

Paraíba (Northeast)

Paraná (South)

Pernambuco (Northeast)

Piauí (Northeast)

Rio de Janeiro (Southeast)

Rio Grande do Norte (Northeast)

Rio Grande do Sul (South)

Rondônia (North)

Roraima (North)

Santa Catarina (South)

São Paulo (Southeast)

Sergipe (Northeast)

Tocantins (North)

**Q4.** What is your marital status? [SA]

Single

Married/Stable Union

Separated/Separated/Divorced

Widow

Another, which one? [OPEN TEXT]

**Q4.** Do you have children? [SA]

Yes

No

[IF YES – Open on the same page]

**Q4a.** How many kids do you have? [Numeric – Range: 1-10]

____ Children

**Q4b.** Do you want to have more children in the future? [SA]

Yes

No

I don't know / I haven't decided yet

**Q4c.** Do you expect to have children in the future? [SA]

Yes

No

I don't know / I haven't decided yet

**Q5.** Are you currently sexually active, i.e., have you had sexual intercourse in the last year? [SA]

Yes

No

**Q6.** Do you have any of the conditions below? [MA]

I have/I had a tubal ligation

I am in the post-menopause period [CLOSE]

I have a vasectomized steady partner

I am against the use of contraceptive methods [CLOSE]

I had surgery to remove the uterus, fallopian tubes, or ovaries (Partial or total) [CLOSE]

I don't fit in the above 5 options

**Q7.** Do you currently use any contraceptive method, that is, any pharmaceutical method, surgical and/or natural to avoid pregnancy? [SA]

Yes [RECRUIT – MAX. 2000 WOMEN AGREE WITH THE REGIONAL AND CLASS DISTRIBUTION SOCIOECONOMIC]

No [SKIP TO QUESTIONNAIRE BLOCK SOCIOECONOMIC AND END – MAX. 1000 WOMEN]

**Q8.** For what reasons do you not currently use any contraceptive method? [SA – Only for Q7 = “No”]

I don't have a partner

I have/am in a same-sex relationship

Gestational desire / I want to get pregnant / I am currently pregnant

The costs of the methods I would like to use are high

I don't know how to use the methods I have access to

Not sure which method is the best to use

I can't get pregnant

I have any health restrictions, which ones? [OPEN] I___I___I

Other reasons, what? [OPEN] I___I___I

**Main questionnaire**

**Q1.** What types of contraceptive methods (avoid pregnancy) do you know? [OPEN –

SUGGEST AUTOFILL AFTER 5 LETTERS]

**Q2.** In addition to those already mentioned, which of these types of contraceptive methods (to avoid pregnancy) do you know? [MA – ONLY CATEGORIES NOT SELECTED IN Q1]

Daily Oral Pill / Oral Contraceptive

Hormonal IUD

Emergency contraceptive/morning-after pill

Hormone Injection

Contraceptive patches/adhesive hormones

Subcutaneous Implants

Male Condom / External Condom

Female Condom / Internal Condom

Copper IUD

Vaginal Ring

Diaphragm / Sponges

Coitus interruptus/ Withdrawal

Calendar-based method / Sex only out of the fertile period

Basal temperature method/body temperature after waking up

Cervical mucus method / Billings method

Use of spermicide / spermicidal creams

Tubal ligation

Partner vasectomy

Another, which one? [OPEN] I___I___I

**Q4.** And of these, which is the current contraceptive method (avoid pregnancy)? [SA]

Daily Oral Pill / Oral Contraceptive

Hormonal IUD

Emergency contraceptive/morning-after pill

Hormone Injection

Contraceptive patches/adhesive hormones

Subcutaneous Implants

Male Condom / External Condom

Female Condom / Internal Condom

Copper IUD

Vaginal Ring

Diaphragm / Sponges

Coitus interruptus/ Withdrawal

Calendar-based method / Sex only out of the fertile period

Basal temperature method/body temperature after waking up

Cervical mucus method / Billings method

Use of spermicide / spermicidal creams

Tubal ligation

Partner vasectomy

Another, which one? [OPEN] I___I___I

**Q5.** How long have you been using the current contraceptive method?[SA]

Less than 6 months ago 1

Between 6 months and 1 year 2

Between 1 year and 3 years 3

Between 3 and 5 years 4

Between 5 and 10 years 5

Between 10 and 15 years 6

Between 15 and 20 years old 7

Between 20 and 30 years 8

More than 30 years ago 9

**Q6.** How often do you use your current contraceptive method? [SA]

Daily

Between 2 and 6 days a week

Weekly

Fortnightly

Monthly

Only after intercourse

Another, which one? [OPEN] I___I___I

**Q7a.** For what reasons do you use [BROUGHT ANSWER FROM Q4]? [MA]

Avoid pregnancy [ do not apply for not sexually active ]

Menstrual flow control

Regularize the menstrual cycle

Hormone control

Improving the skin/fighting acne

It's the same one that a relative / friend uses

Protection against sexually transmitted infections (STIs)

Improve symptoms such as premenstrual tension

Due to some gynecological disease, which one? [OPEN] I___I___I

Others, specify [OPEN] I___I___I

**Q7b.** [if not sexually active] You mentioned that you are not sexually active at the moment, but you use contraceptive methods, please explain in more detail the reasons for this use.

[OPEN]

**Q9**. What/or who influenced your choice to use the current contraceptive method? [MA - APPLY ROTATION]

Physician -> 1

Other health professionals

Relative (Mother/Sister/Grandmother)

Friend

Teacher

Social Networks > Which_________?

Magazines > Which_________?

Advertisements

Internet searches

School / Faculty / Lectures

Partner

Another person, which one? [OPEN] I___I___I

No one influenced my choice

**Q9a.** Your choice to use a contraceptive method was based on which main factor? [SA]

Ease of use/adherence

Availability

More modern method

Method effectiveness

Price

Health benefits

It's the only one I know

It's the only one I have access to / I can buy

I don't want to have more children

Another reason, what? [OPEN] I___I___I

**Q10.** [If Q9 =1] You mentioned that a doctor influenced your decision to use the current contraceptive method, what is this doctor's specialty? [MA]

General Practitioner

Gynecologist/ Obstetrician

Family physician

Another specialty, which one? [OPEN] I___I___I

I don't know / I don't remember the medical specialty

**Q16.** Who pays / paid, or assumes the costs, to acquire the current contraceptive method? [MA]

Myself

My sexual partner

SUS / picked up at the post / public hospital

Family

Others, who? [OPEN] I___I___I

My method has no costs

**Q17.** Who is responsible for choosing my method of use the current contraceptive method? [SA]

The decision is mine alone

I share my decision with my sexual partner

The decision was made in conjunction with my doctor

The decision is solely for my doctor

The decision was taken together with my family

**Q11.** Before using the current contraceptive method have you already used any other contraceptive method? [MA]

Yes

No

**Q11a.** [If = YES in Q11] Which one? [MA]

Daily Oral Pill / Oral Contraceptive

Hormonal IUD

Emergency contraceptive/morning-after pill

Hormone Injection

Contraceptive patches/adhesive hormones

Subcutaneous Implants

Male Condom / External Condom

Female Condom / Internal Condom

Copper IUD

Vaginal Ring

Diaphragm / Sponges

Coitus interruptus/ Withdrawal

Calendar-based method / Sex only out of the fertile period

Basal temperature method/body temperature after waking up

Cervical mucus method / Billings method

Use of spermicide / spermicidal creams

Tubal ligation

Partner vasectomy

Another, which one? [OPEN] I___I___I

**Q11b.** [If Q4 = Q11a] For what reason(s) did you use the contraceptive method same class / same type of contraceptive method? Ex.: Swap pill for another pill or IUD for another IUD [OPEN]

**Q11c**. [If 1 = YES in Q11] For what reason(s) did you switch these contraceptive methods? [MA - APPLY ROTATION]

Fear/Fear of some complication caused by the previous method side effects

Loss of libido

Frequent forgetfulness of daily use

More comfort with the current method

More security with the current method

Doctor's suggestion

Difficult to find in the market/difficulty to maintain

High cost of the contraceptive method

Another, which one? [OPEN] I___I___I

**Q11d.** [If 2 = NO in Q11] For what reason(s) did you not use any method contraceptive before what you currently use? [MA]

Fear/Fear of some complication caused by the contraceptive method side effects

Loss of libido

Frequent forgetfulness of daily use

Doctor's suggestion

Was not sexually active

Pregnancy / recent delivery / breastfeeding

High cost of contraceptives

Another, which one? [OPEN] I___I___I

**Q12.** Do you consider the current contraceptive method your ideal contraceptive method, that is, are you satisfied with your current method? [MA]

Yes

No

**Q12a.** [Q12 = “YES”] For what reason(s) do you consider your current method ideal? [MA]

I feel safe with my current method

I am comfortable with my current method

I don't need to worry about taking my current method

I have better control of my menstrual cycle

I like to see that the method is taking effect

Lower hormone dosage

Does not interfere with my weight

Doesn't change my libido

Another, which one? [OPEN] I___I___I

**Q12b.** [Q12 = 2 “NO”] What would be your ideal contraceptive method? [SA]

Daily Oral Pill / Oral Contraceptive

Hormonal IUD

Morning after pill

Hormone Injection

Adhesive Hormones

Subcutaneous Implants

Male Condom / Condom

Female condom

Copper IUD

Vaginal Ring

Diaphragm / Sponges

Withdrawal

Calendar-based

Basal temperature method/body temperature after waking up

Cervical mucus method / Billings method

Use of spermicide / spermicidal creams

Tubal ligation

Partner vasectomy

Another, which one? [OPEN]

**Q12c**. [Q12 = “NO”] For what reasons do you not use the stated ideal method? [MA - APPLY ROTATION]

This method is very expensive

My doctor has not yet recommended this method to me

I don't have access through the public healthcare system SUS

I cannot use this method because of health complications

Needs discipline/dedication to be effective / to function properly

The method I already use is irreversible

I don't know how to acquire/get this method

Another, which one? [OPEN] I___I___I

**Q13.** In an ideal world, what would be the dosage/frequency you would like to use a contraceptive method? [SA]

Daily, no monthly break

Daily, with monthly break

Weekly

Monthly / 1 time per month

Quarterly / 1 time every 3 months

Annual / 1 time a year

Periods greater than 1 year

Continuous use / I don't want to worry about dosage or frequency

Only when having sex / at the time of sexual intercourse

Another, which one? [OPEN] I___I___I

I don't know

**Q14.** At some point, have you stopped taking your contraceptive method on your own? [SA]

Yes 1

No 2

**Q14a.** [Q14 = 1] For what reason(s) did you stop using your contraceptive method? [MA]

I wanted to get pregnant

Health Complications

Switching contraceptive method

Lack of money

Lack of access to the method in the public system

Side effects

Loss of libido

Weight change

Frequent Forgetfulness

Another, which one? [OPEN] I___I___I

**Q14b.** Thinking about contraception in general, you prefer to keep your menstrual flow monthly, which means, during the pause of the contraceptive method your period would normally come, or you prefer the absence of a period? Why?

I rather keep my period monthly. For what reason?__________

I rather have the absence of monthly periods. For what reason?________

**Q15.** Now, thinking generally, are there any needs you have that are not yet met by the contraceptive methods available to you? If yes, which one? [OPEN]

**Sociodemographic questionnaire**

**S2.** What is your schooling? [SA]

Illiterate / Incomplete Primary I / Incomplete Primary (0 - 4 school years)

Elementary I complete / Elementary II incomplete / Gymnasium incomplete /

Complete Primary (4-9 school years)

Elementary completed / High school incomplete / High school incomplete / Gymnasium

complete (9-12 school years)

Complete high school / Complete high school / Incomplete higher education (≥ 12 school years)

Higher Complete

Post Graduate

**S3.** Adding up all the people who live with you, how much is approximately

your monthly income? [SA] (R$ = BRL)

Between 0 and BRL R$1,100

Between R$1,101 and R$2,200

Between R$2,201 and R$3,300

Between R$3,301 and R$5,500

Between R$5,501 and R$10,100

Between R$10,101 and R$20,200

More than BRL 20,201

Don't know / Prefer not to answer

**S4.** What is your occupation/profession at the moment? [SA]

Registered Professional / Signed Card [Economically active]

Informal full-time work [Economically active]

Part-time informal work [Economically active]

Entrepreneur / Self Employed [Economically active]

Housewife

Unemployed

Just student

Lives on income / Rentista

Another, which one? [OPEN] [Economically active]

I prefer not to answer

**S5.** [If [Economically active]] How many % of your total household income does your income currently represent? [Numeric - Range 0-100%]

____ %

**S6.** Do you have health insurance? [SA]

Yes

Yes, but I choose to seek/acquire my contraceptive method through SUS (public health system)

No
